# *CoreSimul*: a forward-in-time simulator of genome evolution for prokaryotes modeling homologous recombination

**DOI:** 10.1186/s12859-020-03619-x

**Published:** 2020-06-24

**Authors:** Louis-Marie Bobay

**Affiliations:** Department of Biology, University of North Carolina Greensboro, 321 McIver Street, PO Box 26170, Greensboro, NC 27402 USA

**Keywords:** Simulator, Genome evolution, Homologous recombination, Prokaryotes

## Abstract

**Background:**

Prokaryotes are asexual, but these organisms frequently engage in homologous recombination, a process that differs from meiotic recombination in sexual organisms. Most tools developed to simulate genome evolution either assume sexual reproduction or the complete absence of DNA flux in the population. As a result, very few simulators are adapted to model prokaryotic genome evolution while accounting for recombination. Moreover, many simulators are based on the coalescent, which assumes a neutral model of genomic evolution, and those are best suited for organisms evolving under weak selective pressures, such as animals and plants. In contrast, prokaryotes are thought to be evolving under much stronger selective pressures, suggesting that forward-in-time simulators are better suited for these organisms.

**Results:**

Here, I present *CoreSimul*, a forward-in-time simulator of core genome evolution for prokaryotes modeling homologous recombination. Simulations are guided by a phylogenetic tree and incorporate different substitution models, including models of codon selection.

**Conclusions:**

*CoreSimul* is a flexible forward-in-time simulator that constitutes a significant addition to the limited list of available simulators applicable to prokaryote genome evolution.

## Background

Many bioinformatic tools rely on genome simulators to infer parameters or to validate new methodologies [1–4]. Although a large diversity of genome simulators have been released, such tools are usually designed for specific tasks and are not adapted to all types of analyses [5–13]. Specifically, relatively few simulators have been implemented to simulate the evolution of prokaryote genomes [1, 4, 14], whose biology differs substantially from eukaryotic organisms [15]. One key difference is the inability of prokaryotes to engage in meiotic recombination. Instead, prokaryotes engage in homologous recombination through gene conversion, which consists in the non-reciprocal transfer and replacement of a sequence by an homologous one, typically leading to the exchange of short sequences [16–18].

Recent analyses are suggesting a prevalent role of homologous recombination in prokaryotes and the vast majority of bacterial species appears to be impacted by this process, indicating that simulation frameworks incorporating homologous recombination are needed [16, 19–21]. Indeed, the presence of homologous recombination prevents the application of purely clonal frameworks to simulate prokaryote evolution, as well as methods assuming true sexual reproduction [22]. Moreover, the strong selective constraints typically acting on bacterial genome evolution [23, 24] must be taken into account in order to simulate realistic genome datasets.

Multiple coalescent-based simulators have been implemented and several can be applied to simulate prokaryote evolution with homologous recombination [4, 25, 26]. Although coalescent-based simulators offer interesting properties to simulate the evolution of genomic sequences, forward-in-time simulators present alternative qualities that can be better suited for certain tasks. In particular, coalescent-based simulators are designed to simulate sequences under a neutral model of evolution [27], an assumption that is likely violated in prokaryotes, where adaptive evolution could be predominant [28, 29]. Although multiple forward-in-time simulators have been implemented [1, 27], they are rarely adapted to simulate the evolution of prokaryote genomes.

Here I present *CoreSimul*, an efficient forward-in-time simulator to model prokaryotic genome evolution with homologous recombination and selection along phylogenetic trees (https://github.com/lbobay/CoreSimul).

## Implementation

*CoreSimul* generates a set of prokaryote genomes based on a phylogenetic tree. *CoreSimul* allows to simulate both the core genome—the set of genes conserved across all genomes—and the accessory genome—the set of genes shared by a subset of genomes—of a population or a species. The *CoreSimul* process starts by generating a random core genome sequence of length *L* and with a GC-content *GC* specified by the user. Alternatively, an input genome can be directly provided by the user. The sequence is assumed to represent a nucleotide concatenate of protein coding genes without intergenic DNA. This sequence is then evolved in silico following a branching process respecting the topology of the input tree. The rate of substitutions *m* is based on the branch length of the input tree, and this rate can be modified by the user with a rescaling coefficient (e.g. a rescaling coefficient of 0.5 will reduce the length of all branches by half). In order to mimic the effect of purifying selection acting on coding sequences, the sequence can be evolved with different substitution rates across codon positions: the relative rates of the three codon positions can be specified by the user (see user manual for recommendations). When specified, the relative rate of substitution across codon positions does not change the overall rate of substitution, which is imposed by the branch length of the input tree and the rescaling coefficient. In addition, several substitution models can be specified: Juke and Cantor (JC69), Kimura 2-parameters (K2P), Kimura 3-parameters (K3P) or General Time Reversible (GTR), in which case, the substitutions transition/transversion ratio *κ* or other parameters must be specified. Finally, the genomes are evolved with a recombination rate *ρ*, which is defined relative to the substitution rate *m*. Homologous recombination events are internal to the simulated dataset and no imports from external sources are modelled (unless gene gains are allowed). Note that *CoreSimul* makes a clear distinction between homologous recombination, which consists in the replacement of a sequence by an homologous one present in the simulated dataset, from horizontal gene transfer, which consists in the gain of a new sequence external to the simulated genomes (see below). In effect, recombination leads to the exchange of single nucleotide polymorphisms while gene gains lead to the acquisition of new sequences.

**Fig. 1:**
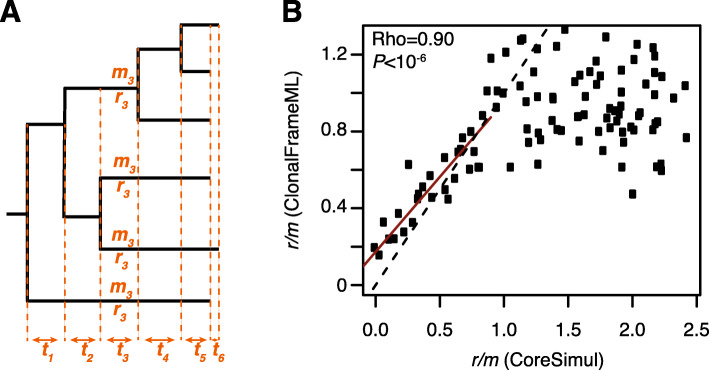
**a**. Schematic representation of the *CoreSimul* process. The tree is divided in multiple time segments with the same number of overlapping branches. Only branches in the same time segment can recombine with one another. For each branch in the time segment, an average number *m*_*t*_ of mutations is introduced following a Poisson distribution and an average number *r*_*t*_ of recombination events is introduced—also following a Poisson distribution—between the branches with *r*_*t*_ = *ρ.m*_*t*_ with *ρ* the recombination rate. Mutation events and recombination events are introduced in a random order in the different branches of the time segment, simultaneously. **b**. Comparison between *CoreSimul* simulations and recombination rate predictions of *ClonalFrameML*. The black dashed line represents the theoretical expectation between *CLonalFrameML* predictions and the recombination rates in the simulated dataset. The red line represents the observed linear regression between the simulated *r/m* values and the *r/m* values predicted by *ClonalFrameML* (note that only the data points for *r/m* ≤ 1 were used for the regression)

In order to mimic more realistic conditions, the different sequences present at any given time are evolved simultaneously and only sequences overlapping in time are allowed to recombine with one another (i.e. recombination with ancestral sequences is not allowed). Concretely, the phylogenetic tree is divided in multiple “time segments” of overlapping branches (Fig. 1a). For each time segment *t*, each sequence receives a number of mutations *M*_*t*_ and a number of recombination events *R*_*t*_ defined by a Poisson process of mean *m.l* and *ρ.l*, respectively, with *l* the length of the branch in the time segment, *m* the mutation rate and *ρ* the recombination rate. The mutation and recombination events are then introduced at random in different sequences of the time segment: a random sequence of the time segment is pulled and a mutation event or a recombination event is introduced randomly (this step is repeated until all the mutation and recombination events specific to each sequence have been introduced). The donor sequence of each recombination event is pulled randomly from the set of sequences in the time segment. The position of each recombination event is chosen at random along the sequence and its size is defined by a geometric distribution of mean *δ* specified by the user (genomes are assumed to be linear).

During the simulation the number of nucleotide polymorphisms (SNPs) exchanged by each recombination event is recorded to generate the statistic *η*, which represents the average number of polymorphic alleles exchanged by recombination. Using this statistic, the effective recombination rate *r/m* is defined from the relationship *r/m = ρ.η.δ* as in [2] and is returned to the user at the end of the simulation. The effective recombination rate *r/m* is frequently used to measure recombination rates and represents the number of polymorphisms exchanged by recombination relative to the number of polymorphisms introduced by mutation.

*CoreSimul* further offers the possibility to simulate uneven rates of homologous recombination along the genome as a function of sequence divergence. Multiple works have experimentally determined that the frequency of homologous recombination decreases with sequence divergence following a log-linear relationship [30–36]. As a result, it is predicted that homologous sequences with higher sequence identity will be much more likely to recombine than more divergent sequences. When specified by the user, *CoreSimul* introduces a biased probability of homologous recombination using the relationship *p = 10*^*-πΦ*^*,* with *p* the probability to recombine, *π* the sequence divergence and *Φ* the slope of the relationship between sequence divergence and the frequency of homologous recombination [33]. The coefficient *Φ* is species-specific and has only been determined for several species [33]. Thus, by default, *CoreSimul* uses a parameter of *Φ =* 18.1, as experimentally inferred for *Streptococcus pneumoniae*, which is intermediate relative to other species with known *Φ* values such as *Bacillus subtilis* and *Escherichia coli* [33].

In addition, rates of gene gains and losses can be specified to simulate the evolution of accessory genes. In the *CoreSimul* framework, gene gains are modeled as external horizontal gene transfers, independent from homologous recombination events, which are only modeled as internal events. The rates of gene gains and losses are specified as a function of the substitution rate following a Poisson distribution (i.e. proportional to branch length). Assuming purifying selection, entire genes can be gained or lost but not fragments thereof. Simulating genomes with gene gains and/or losses results in a genome composed of core genes and accessory genes: Core genes are those genes present in all the genomes of the dataset, while accessory genes are those genes only found in a fraction of the genomes. During the simulation, homologous accessory genes are free to engage in homologous recombination with one another if the sequence is present in both the donor and the recipient genomes. In the case where a recombining fragment overlaps an accessory and a core gene, the accessory portion of the gene will not be transferred from the donor to the recipient genome if this sequence is absent in the recipient genome (i.e. only the fragment of the core gene will be transferred). Consequently, homologous recombination will not introduce partial indels from one genome to another but will transfer entire genes or lead to the deletion of entire genes if both extremities of the recombining fragment are located within homologous sequences shared by both the donor and the recipient genomes. Note that if no gene gains or losses are specified, *CoreSimul* will only simulate the evolution of the core genome.

*CoreSimul* is implemented in Python 3.7 but can also run on Python 2.7 without modification. It requires the Python library NumPy. No other dependencies are required. The parameters of the simulation must be provided in a control file. *CoreSimul* is compatible with Mac and Linux operating systems. *CoreSimul* can be freely downloaded at https://github.com/lbobay/CoreSimul. The program also includes a user manual with detailed information, recommendations and examples.

## Results and discussion

To test *CoreSimul*, we simulated the evolution of a genome following the topology and branch lengths of a previously published tree [24]; the tree was originally built using the core genome of 34 genomes of *Acinetobacter pittii* using RAxML v8 [37]. The genomes were simulated with a length of 100,000 bp, a GC-content of 50%, *κ* = 1, and no selection (identical substitution rates across codon positions). The tree was rescaled with parameter 0.05 to reduce the number of polymorphisms in the simulated alignments. One hundred simulations were conducted with recombination rates *ρ* varying from 0 to 5. The average recombination tract length was set at *δ* = 100 bp across all simulations. The 50 simulated datasets were then analyzed with *ClonalFrameML* [3] with the same input tree and with default parameters. We observed nearly identical values between the recombination rates generated with *CoreSimul* and the rates predicted by *ClonalFrameML* when recombination rate was low (Fig. 1b; Spearman’s Rho = 0.90,*P* < 10^− 6^ for *r/m* ≤ 1). A very similar relationship (Supplementary Figure 1; Spearman’s Rho = 0.89, *P* < 10^− 6^ for *r/m* ≤ 1) was observed when the sequences were evolved with more realistic parameters that closely match this species, i.e. GC = 45%, *κ* = 1.6 and different substitution rates across codon positions (0.15, 0.07 and 0.78 for codon positions 1, 2 and 3, respectively). Note that for higher recombination rates (*r/m* > 1 in this case), *ClonalFrameML* tends to substantially underestimate recombination rates (Fig. 1b) as reported by the authors of the program [3]. In addition, we found stronger discrepancies between our simulations and the *r/m* ratios inferred by *ClonalFrameML* for datasets simulated with higher substitution rates.

In addition, we conducted multiple verifications to ensure that the entered parameters were simulated as expected: The GC-content, the alignment length and the transition/transversion ratio *κ* (or the rates for other substitution models) were systematically found to match the simulation parameters. We also observed that the polymorphisms at the different codon positions matched the relative substitution frequency specified across first, second and third codon positions. Finally, we could retrieve the same tree topology as the input tree when building the phylogenetic tree from the simulated sequences with RAxML v8 [37]. However, tree topologies were observed to differ from the input tree when simulated with higher recombination rates, which is expected due to the increase of homoplasies in the sequences when simulated with higher recombination rates. Overall, these different tests confirmed that the sequences were simulated as expected.

## Conclusions

*CoreSimul* is a forward-in-time simulator, specifically built to simulate prokaryote evolution with homologous recombination and negative selection across codon positions. Such a tool can be used to infer parameters or to test other bioinformatic tools. In addition, because it is based on a phylogenetic framework, *CoreSimul* incorporates population structure information, which can be applied to population models and phylogenetic analyses. Although many genome simulators have been implemented, *CoreSimul* presents several key differences relative to existing tools: i) it does not rely on the coalescent but uses a tree-guided simulation framework, ii) it is specifically designed to simulate the evolution of prokaryotic genomes with a prokaryote-specific model of homologous recombination, iii) it can be run with customized parameters and models of substitution, iv) it offers the possibility to model gene gains and gene losses, thereby simulating the evolution of core and accessory genes and v) it can model varying rates homologous recombination as a function of sequence divergence. Overall, *CoreSimul* constitutes a substantial addition to the current list of genome simulators. This addition is particularly valuable considering the limited number of forward-in-time simulators currently available to model prokaryote genome evolution.

### Availability and requirements

**Project name**: CoreSimul.

**Project home page:** https://github.com/lbobay/CoreSimul

**Operating system(s):** MacOS, Linux.

**Programming language:** Python.

**Other requirements:** Numpy library.

**License:** MIT License.

**Any restrictions to use by non-academics:** None.

## Supplementary information

**Additional file 1: Figure S1.** Comparison between *CoreSimul* simulations with selection and recombination rate predictions of *ClonalFrameML*. Simulations were run with parameters that closely match the sequence parameters of *A. pittii*: GC = 45%, transition/transversion ratio *κ* = 1.6, relative substitution rates of codon positions: 0.15, 0.07 and 0.78 for codon positions 1, 2 and 3, respectively. The black dashed line represents the theoretical expectation between *ClonalFrameML* predictions and the recombination rates in the simulated dataset. The red line represents the observed linear regression between the simulated *r/m* values and the *r/m* values predicted by *ClonalFrameML* (note that only the data points for *r/m* ≤ 1 were used for the regression).

## Data Availability

The scripts and datasets used for this analysis are freely accessible on https://github.com/lbobay/CoreSimul.
