# Supplementary figures and images for: CoreSimul: a forward-in-time simulator of genome evolution for prokaryotes modeling homologous recombination
Source: BMC Bioinformatics. 2020 Jun 24;21:264. doi: 10.1186/s12859-020-03619-x (PMC7315543; doi:10.1186/s12859-020-03619-x)

$r/m$  (ClonalFrameML)

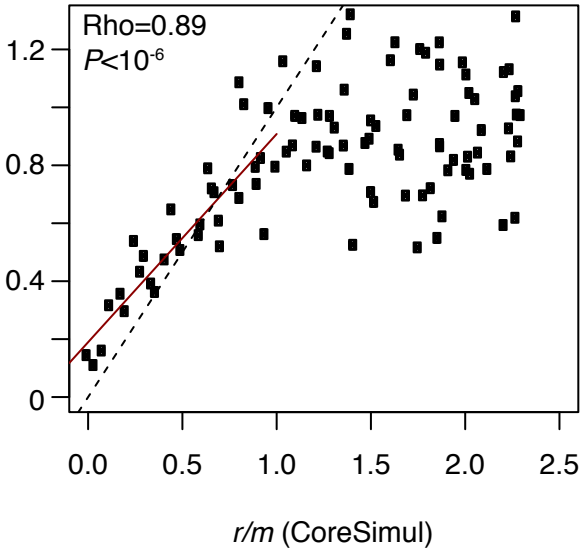

Supplement: Supplementary file 1 — Additional file 1: Figure S1. Comparison between CoreSimul simulations with selection and recombination rate predictions of ClonalFrameML. Simulations were run with parameters that closely match the sequence parameters of A. pittii: GC = 45%, transition/transversion ratio κ = 1.6, relative substitution rates of codon positions: 0.15, 0.07 and 0.78 for codon positions 1, 2 and 3, respectively. The black dashed line represents the theoretical expectation between ClonalFrameML predictions and the recombination rates in the simulated dataset. The red line represents the observed linear regression between the simulated r/m values and the r/m values predicted by ClonalFrameML (note that only the data points for r/m ≤ 1 were used for the regression). [file 12859_2020_3619_MOESM1_ESM.pdf]
